# Cupping therapy and chronic back pain: systematic review and meta-analysis

**DOI:** 10.1590/1518-8345.2888.3094

**Published:** 2018-11-14

**Authors:** Caroline de Castro Moura, Érika de Cássia Lopes Chaves, Ana Carolina Lima Ramos Cardoso, Denismar Alves Nogueira, Hérica Pinheiro Corrêa, Tânia Couto Machado Chianca

**Affiliations:** 1 Universidade Federal de Minas Gerais, Escola de Enfermagem, Belo Horizonte, MG, Brazil.; 2 Universidade Federal de Alfenas, Escola de Enfermagem, Alfenas, MG, Brazil.; 3 Universidade Federal de Alfenas, Instituto de Ciências Exatas, Alfenas, MG, Brazil.

**Keywords:** Review, Chronic Pain, Back Pain, Cupping Therapy, Meta-Analysis, Nursing

## Abstract

**Objectives:**

to evaluate the evidence from the literature regarding the effects of cupping therapy on chronic back pain in adults, the most used outcomes to evaluate this condition, the protocol used to apply the intervention and to investigate the effectiveness of cupping therapy on the intensity of chronic back pain.

**Method:**

systematic review and meta-analysis carried out by two independent researchers in national and international databases. Reference lists of systematic reviews were also explored. The quality of evidence was assessed according to the Jadad scale.

**Results:**

611 studies were identified, of which 16 were included in the qualitative analysis and 10 in the quantitative analysis. Cupping therapy has shown positive results on chronic back pain. There is no standardization in the treatment protocol. The main assessed outcomes were pain intensity, physical incapacity, quality of life and nociceptive threshold before the mechanical stimulus. There was a significant reduction in the pain intensity score through the use of cupping therapy (p = 0.001).

**Conclusion:**

cupping therapy is a promising method for the treatment of chronic back pain in adults. There is the need to establish standardized application protocols for this intervention.

## Introduction

Chronic back pain causes physical, emotional and socioeconomic changes^1^ ^-^ ^3^ and, consequently, high use of medicines and health resources^4^. The search for demedicalization leads to an increasing use of integrative and complementary practices, such as Traditional Chinese Medicine (TCM) resources, to complement pain-related allopathic care^5^. Cupping therapy is one of the recommended TCM therapies for chronic pain reduction^6^. It involves the application of cups of different materials^7^ in an acupoint or area of pain by means of heat or vacuum apparatus^8^.

The effect on pain reduction has not yet been fully elucidated^9^, but different mechanisms of action, based on several assumptions^10^, are attributed to cupping therapy, such as the metabolic, neuronal hypotheses^9^ ^,^ ^11^ and TCM^12^. Evidence of the efficacy of this intervention is limited because of the lack of high quality, well-delineated randomized controlled trials (RCTs)^6^ that result in validated and efficient protocols for the treatment of chronic back pain. Therefore, this study aims to evaluate the literature evidence regarding the effects of cupping therapy on chronic back pain in adults compared to sham, active treatment, waiting list, standard medical treatment or no treatment, outcomes most commonly used to assess this condition, the protocol used to apply the intervention and subsequently investigate the effectiveness of cupping therapy on the intensity of chronic back pain.

## Method

A systematic review of the literature was performed, followed by meta-analysis, used to determine the intensity of back pain in adult clients. The study was based on the criteria of the Preferred Reporting Items for Systematic Reviews and Meta-Analyzes (PRISMA Statement)^13^.

The PICO (P - population; I - intervention; C - comparison; O - outcomes)^14^ guided the elaboration of the guiding question: “What are the effects of cupping therapy on adults with chronic back pain?”

The search strategy, carried out by two independent reviewers from June 2017 to May 2018 was based on the following databases: Medical Literature Analysis and Retrieval System Online (MEDLINE) via the US National Library of Medicine National Institutes of Health (PUBMED), Web of Science, The Cumulative Index to Nursing and Allied Health Literature (CINAHL), Physiotherapy Evidence Database (PEDro), Embase, Scopus, as well as databases indexed in the Virtual Health Library (VHL), such as Latin American & Caribbean Health Sciences Literature (LILACS) and the National Information Center of Medical Sciences of Cuba (CUMED). Reference lists of systematic reviews were also explored in the search for relevant studies related to the guiding question.

The terms, controlled and free, were combined by means of the Boolean operators OR and AND as follows: (“Back Pain” OR “Low Back Pain” OR “Sciatica” OR “Chronic Pain” OR “Musculoskeletal Pain” OR Myalgia OR “Neck Pain” OR “Low Back Pains” OR “Musculoskeletal Pains” OR “Muscle Pain” OR “Neck Pains” OR “Cervical Pain” OR “Cervical Pains” OR Lumbago OR “lumbar pain”) AND (“cupping therapy” OR cupping OR cups).

The eligibility criteria for the selection of articles were: RCT with adults (18 years or older); chronic pain (for three months or more)^15^ in at least one of the segments of the spine (cervical, thoracic and/or lumbar); use of cupping therapy (dry, wet, massage, flash)^7^ compared to one or more of the following groups: sham, active treatment, waiting list, standard medical treatment, or no treatment. We excluded studies that did not present online abstract in full for analysis, those that were not located by any means and studies with pregnant women.

In order to collect the information from the selected studies, we used an adapted form^16^ in accordance with the recommendations of the Revised Standards for Reporting Interventions in Clinical Trials of Acupuncture (STRICTA)^17^ and the classifications of cupping therapy^7^ ^,^ ^18^.

The following data were extracted: article identification (title, author (s)/training area, journal, year of publication, study country/language); objectives; methodological characteristics (design, sample size and loss of follow-up; inclusion and exclusion criteria); clinical data (number of patients by sex, mean age, diagnosis, duration of symptoms); description of interventions in the follow-up groups (number of sessions, duration of treatment, type of technique applied (dry, wet, flash or massage cupping), application device, time of stay of the device, suction method (manual, fire, automatic-electric)/suction strength (light, medium, strong or pulsating)^18^; peculiarities of the intervention; application points; training area of the professional who carried out the intervention; years of experience in the area); outcomes and methods of evaluation (number of evaluations, intervals between them, measurement tools); data analysis; main results; and study findings.

The methodological quality of eligible studies was assessed using the Jadad scale^19^, which is centered on internal validity. The questions have a yes/no answer option with a total score of five points: three times one point for the yes responses and two additional points for appropriate randomization and concealment of allocation methods. Two independent reviewers conducted the evaluation, and a third investigator was consulted to solve possible disagreements.

Data analyzes were performed using Stata SE/12.0 statistical software. The absolute difference between means with 95% confidence intervals was selected to describe the mean differences between the treated and control groups in the evaluation performed shortly after treatment. P-value <0.05 was considered as statistically significant. Potential heterogeneity among the studies was examined using Cochran Q^20^ and I^2(^ ^21^ statistics. Since there was statistical significance in the test for heterogeneity of the results (p <0.05) and the calculated value of I^2^ suggested a moderate to high heterogeneity (67.7%)^21^, the random effects model was adopted for the analysis.

## Results

**Figure 1:**
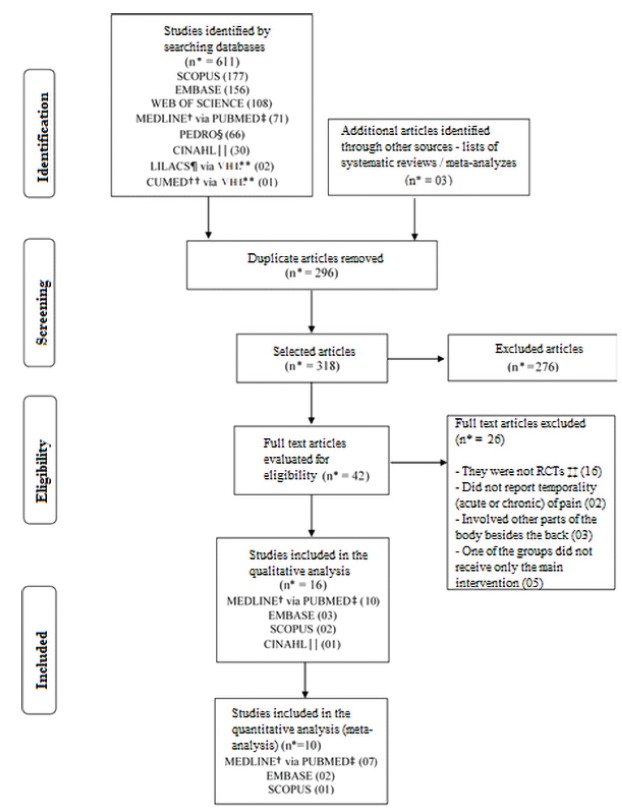
Flowchart of literature search and selection process. Belo Horizonte, MG, Brazil, 2018

A total of 614 studies were found in electronic and manual searches. Of these, 296 were removed from the list because they were duplicates. After reviewing titles and abstracts, 265 studies were excluded and 53 remained for analysis of the full text. Of these, 11 studies were not found (online, via bibliographic switching or direct contact with authors) and 26 articles were excluded. Finally, 16 articles remained in the review for the synthesis of the qualitative analysis and 10 articles entered the quantitative analysis (Figure 1).

*n - Number of articles; †MEDLINE - Medical Literature Analysis and Retrieval System Online; ‡PUDMED - US National Library of Medicine National Institutes of Health; §PEDRO - Physiotherapy Evidence Database; ||CINAHL - The Cumulative Index to Nursing and Allied Health Literature; ¶LILACS - Latin American and Caribbean Health Sciences Literature; **VHL - Virtual Health Library; ††CUMED - National Information Center of Medical Sciences of Cuba; ‡‡RCT - Randomized Clinical Trial

All articles selected were published in English language and were conducted in Germany^9^ ^,^ ^22^ ^-^ ^27^, Taiwan^28^ ^-^ ^30^, Iran^31^ ^-^ ^33^, South Korea^34^ ^-^ ^35^ and in Saudi Arabia^36^. Participants were a total of 1049 people, aged between 18 and 79 years, of whom 519 were in the groups receiving the experimental therapy and 530 in the control groups (sham, waiting list, standard medical treatment/active treatment or no treatment). Of these, all had chronic pain conditions^15^, being the cervical spine/neck the most affected area^9^ ^,^ ^23^ ^-^ ^27^ ^,^ ^29^ ^,^ ^34^, followed by the lumbar region^22^ ^,^ ^28^ ^,^ ^30^ ^-^ ^33^ ^,^ ^35^ ^-^ ^36^. Two other studies^31^ ^,^ ^33^, although they did not make clear the temporality of the pain, were selected because this information could be inferred with great accuracy.

**Figure 2:**
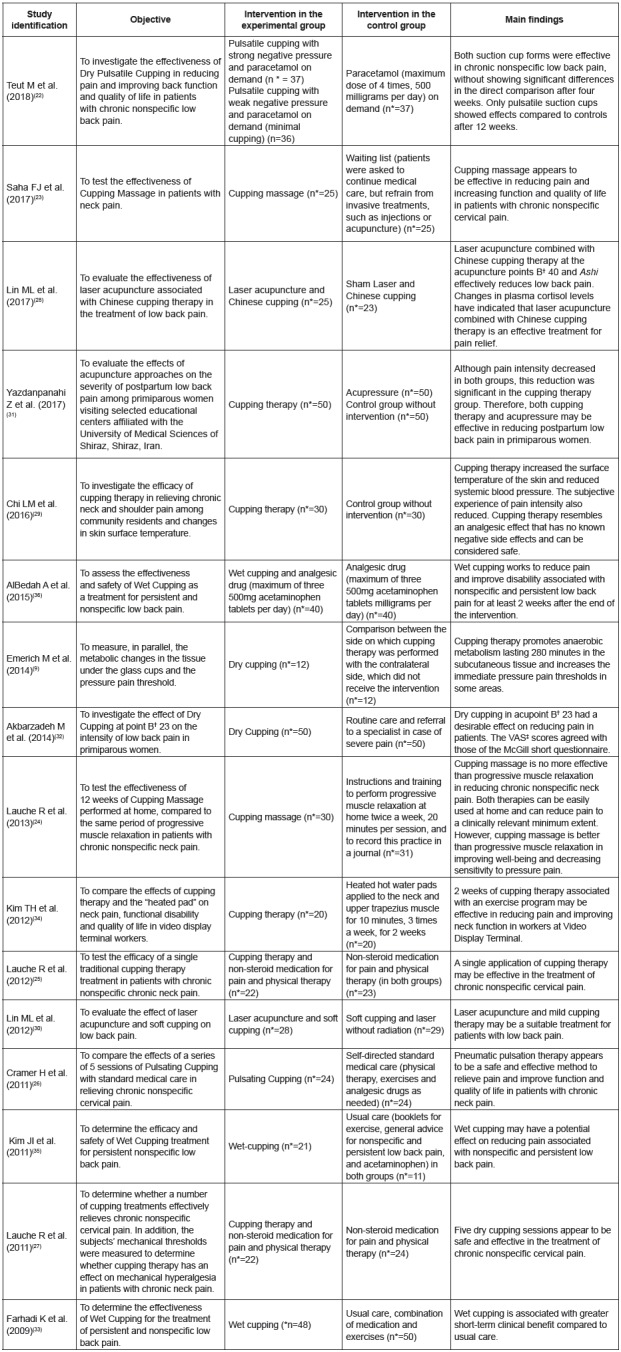
Characterization of the studies regarding the applied intervention, Belo Horizonte, MG, Brazil, 2018 (n=16)

The characterization of the studies regarding the objective, the interventions applied in the experimental and control groups, and the main findings are presented in Figure 2. 

*n - Number of participants; †B - Bladder; ‡VAS - Visual Analogue Scale.

Regarding the methodological quality of the RCTs, all reported the random sequence generation method and in only one study^9^ this process was not appropriate. In another study^30^ there is not enough information to infer this information. Only in four RCTs^22^ ^,^ ^24^ ^,^ ^28^ ^-^ ^29^ ^)^ there was a description of masking and in only two^22^ ^,^ ^28^ this was considered appropriate. Loss of follow-up was not described in only one RCT^29^.

Therefore, 6.25% (n = 1) of the studies^9^ scored one on the Jadad score; 12.5% (n = 2)^29^ ^-^ ^30^ scored two; 62.5% (n=10)^23^ ^,^ ^25^ ^-^ ^27^ ^,^ ^31^ ^-^ ^36^ scored three; 12.5% (n=2)^22^ ^,^ ^24^ score four; and 6.25% (n=1)^28^ scored five points.

**Figure 3:**
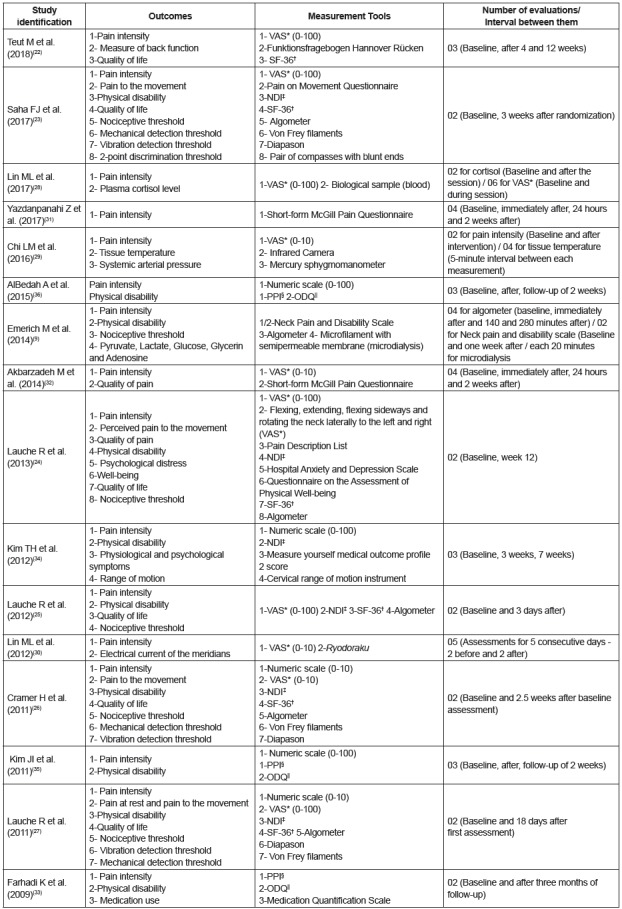
Evaluated outcomes, measurement tools, number of evaluations and interval between them. Belo Horizonte, MG, Brazil, 2018. (n=16)

The studied outcomes, the measurement tools, the number of evaluations and the interval between them are described in Figure 3.

*VAS - Visual Analogue Scale; †SF-36 - Short Form 36 Health Survey Questionnaire; ‡NDI - Neck Disability Index; §PPI- McGill Present Pain Intensity questionnaire; ||ODQ - Oswestry Disability Questionnaire

The most evaluated outcomes were pain intensity (100%; n=16)^9^ ^,^ ^22^ ^-^ ^36^, followed by Physical disability (62.5%; n=10)^9^ ^,^ ^23^ ^-^ ^27^ ^,^ ^33^ ^-^ ^36^, quality of life (37.5%; n=6)^22^ ^-^ ^27^ and nociceptive threshold before the mechanical stimulus, by means of an algometer (37.5%; n=6)^9^ ^,^ ^23^ ^-^ ^27^.

The number of evaluations ranged from two (baseline and after treatment) to 18. Three studies performed evaluations between sessions^9^ ^,^ ^28^ ^-^ ^29^; and 13 studies performed follow-up evaluations after the end of the treatment, ranging from two days to three months ^9^ ^,^ ^22^ ^-^ ^23^ ^,^ ^25^ ^-^ ^27^ ^,^ ^30^ ^-^ ^36^ (Figure 3).

**Figure 4:**
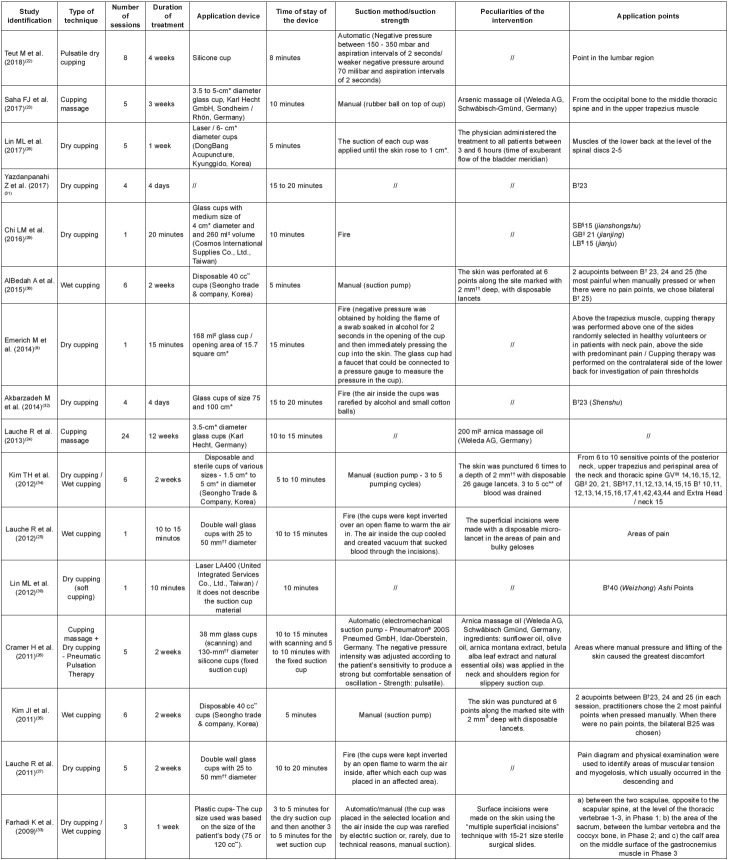
Intervention protocol. Belo Horizonte, MG, Brazil, 2018 (n=16)

The characteristics of the intervention protocol were based on the recommendations of the Revised Standards for Reporting Interventions in Clinical Trials of Acupuncture (STRICTA)^17^ and in the classifications of cupping therapy^7^ ^,^ ^18^, which are described in Figure 4.

*cm - Centimeter; †B - Bladder; ‡ml - Milliliter; §SB - Small bladder; ||GB - Gallbladder; ¶LB - Large bladder; **cc - Cubic centimeter; ††mm - Millimeter; ‡‡GV - Governing Vessel

The intervention was predominantly applied by physicians (31.25%; n=5)^22^ ^,^ ^25^ ^-^ ^28^ ^,^ ^34^; followed by nurses (18.75%; n=3)^22^ ^,^ ^29^ ^,^ ^32^ and pharmacists (6.25%; n=1)^32^. And 25% of the studies (n=4)^9^ ^,^ ^23^ ^,^ ^35^ ^-^ ^36^ reported that the intervention was performed by a therapist, without specifying the training area.

Only 18,75% of the studies (n = 3) presented the time of experience of the professional who performed the intervention, from three^35^ ^-^ ^36^ to four years^34^; 37.5% of the studies (n=6)^9^ ^,^ ^22^ ^-^ ^25^ ^,^ ^27^ informed only that the intervention had been performed by experienced or trained professionals, but did not mention the time of training.

Of the 16 articles selected for the systematic review, 10 entered for meta-analysis that investigated the effectiveness of cupping therapy on pain intensity. All of them approached the outcome in two comparison groups (experimental and control), in evaluations performed before and immediately after the treatment. Five studies^9^ ^,^ ^22^ ^,^ ^29^ ^,^ ^35^ ^-^ ^36^ did not enter because they did not have enough data for this analysis and one study^33^ performed the evaluation only three months after the end of treatment.

**Figure 5:**
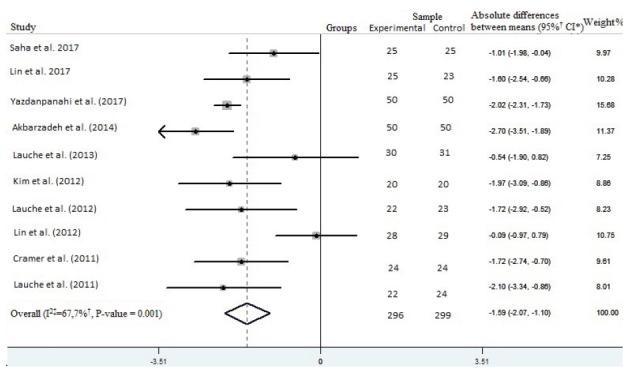
Forest plot of the pain intensity score. Belo Horizonte, MG, Brazil, 2018

The results of the meta-analysis showed that cupping therapy was more effective in reducing pain compared to the control group (absolute difference between means: -1.59, [95% Confidence Interval: -2.07 to -1.10]; p = 0.001), with moderate to high heterogeneity (I^2^ = 67.7%, p = 0.001) (Figure 5).

*CI - Confidence interval; †% - Percentage; ‡I^2^ - Measurement of heterogeneity

## Discussion

Cupping therapy has shown positive results on chronic back pain in adults, not only in behavioral variables of pain, but also in physiological parameters in the majority of RCTs evaluated in this study, which contributes to the consolidation of its use in the treatment of this clinical condition in the study population.

Regarding methodological quality, most studies^23^ ^,^ ^25^ ^-^ ^27^ ^,^ ^31^ ^-^ ^36^ ^)^ obtained a median score (three) according to the Jadad scale^19^. This score can be justified by the lack of masking of RCTs.

It is not feasible to conceal evaluation and intervention methods in cupping therapy^22^, since the marks left by the suction cups are often visible and may persist for several days, making it difficult to perform a masking process^27^. Only one study^28^ achieved masking properly; however, it was true only for volunteers who received laser therapy, an intervention used concomitantly with cupping therapy, where sham laser acupuncture was performed by applying the same procedure in one of the groups, but without energy. In a second study^24^, there is a description that the masking was applied to the evaluator of the results; however, the application of suction cups causes marks (ecchymoses, petechiae) and one of the evaluated outcomes was the pain threshold, using the algometer; for this evaluation, as the area must be naked, the marks on the skin make this kind of masking impossible. Finally, in another study^22^, the majority of participants in the minimal cupping group (84%) was able to identify the allocation after four weeks, whereas in the cupping group 55% identified the allocation.

Regarding the evaluated outcomes, pain intensity predominated, which was measured mostly by means of the Visual Analogue Scale (VAS)^22^ ^-^ ^25^ ^,^ ^27^ ^-^ ^30^ ^,^ ^32^ and the Numerical Scale^26^ ^,^ ^34^ ^-^ ^36^, followed by the Neck Pain and Disability Scale^9^, by the short version of the McGill Pain Questionnaire^31^, and by the Present Pain Intensity Scale^33^.

Although there are variations, the VAS usually consists of scores of 0-10 or 0-100, the extreme left being described as no pain and the extreme right as the worst possible pain; the numerical scale has a numerical rating of 0-10, 0-20 or 0-100. These scales can be classified as: painless (0), mild (1-3), moderate (4-6), and severe (7-10), and are frequently used in patients with chronic musculoskeletal pain^37^. In addition, some researchers^38^ ^-^ ^40^ have pointed to these two scales as the gold standard for assessing pain intensity, these being the instruments most used when evaluating adults, both in clinics and research.

Physical disability was the second most approached outcome, measured by means of the Neck Disability Index (NDI)^23^ ^-^ ^27^ ^,^ ^34^, of the Oswestry Disability Questionnaire (ODQ)^33^ ^,^ ^35^ ^-^ ^36^ and the Neck Pain and Disability Scale^9^. In fact, the severity and chronicity of back pain are associated with severe functional limitations^37^ that imply limitations in activities of daily living^41^.

In addition, patients with chronic diseases, who require continuous treatment over a long period, present important changes in quality of life^42^, being another important outcome to be evaluated, as occurred in six studies, through the Short Form 36 Health Survey Questionnaire (SF-36)^22^ ^-^ ^27^.

Finally, the physiological parameter most evaluated in the studies was the nociceptive threshold before the mechanical stimulus, by means of a pressure algometer^9^ ^,^ ^23^ ^-^ ^27^. It is known that individuals who have pain in the spine have higher nociceptive sensitivity compared to healthy people^43^. However, this is still considered a subjective variable, since it is the patient who determines his/her pain threshold. In fact, when the evaluation process is more related to the symptoms, such as subjective phenomena, especially pain, than to physical or laboratory results, self-assessment is considered the most reliable indicator of the existence of pain^44^. Thus, the necessary information to carry out its evaluation has its origin in the individual’s report^45^, who is the primary source of the assessment.

The systematized analysis of cupping therapy application methods showed that there is no standardization in the treatment protocol for chronic back pain. However, recent efforts have been made to standardize the cupping therapy procedure in general^46^ and specifically for chronic back pain, since the most appropriate type of technique, duration of treatment, number of sessions, devices, time of application, method and suction strength and application points have not been determined.

It can be observed, however, that the most applied technique was dry cupping, specifically for the lumbar^22^ ^,^ ^28^ ^,^ ^30^ ^-^ ^32^ and cervical regions^9^ ^,^ ^27^ ^,^ ^29^ ^,^ ^34^. This modality allows the stimulation of the acupoints in the same way as the acupuncture needles^47^. Researchers^18^ suggest that laceration of the skin and capillaries, promoted by wet cupping, may act as another nociceptive stimulus that activates the descending inhibitory pathways of pain control^18^, thus helping to treat chronic musculoskeletal conditions^35^. However, risk for infection, vasovagal attacks and scars are the disadvantages of this method^18^. Still, compared to cupping massage, authors^47^ emphasize that dry cupping has a greater analgesic effect, since the use of lubricants can reduce the friction between the edge of the cup and the skin, a fact corroborated by some authors^24^ who used arnica oil for the realization of cupping massage.

Despite the variability in the application of the intervention, it was possible to identify that, on average, the cupping therapy was applied in 5 sessions, with permanence of the cups in the skin for around 8 minutes, and interval of three to four days between the applications. According to some researchers^27^, at least five sessions are required for any significant effects of cupping treatment to appear, in addition to ensuring the feasibility of the RCT. Moreover, authors^47^ recommend that the cups should be left on the skin for 5 to 10 minutes or more, which culminates in the appearance of residual marks after treatment as a result of the rupture of small blood vessels that are painless and disappear between 1 and 10 days^12^. Therefore, an interval between sessions is necessary in order to allow the reestablishment of the cutaneous and subcutaneous tissues.

Regarding the application cups, the disposable ones are preferable a high-level sterilization or disinfection process is required prior to reuse, since the pressure exerted may cause extravasation of blood and fluids from the skin^46^. Nowadays, cupping therapy has increasingly been performed with plastic cups^47^. The size of the cups varies according to the place of application, but it is often applied in places with abundant muscles, such as the back^48^.

Regarding the suction method to create negative pressure, the use of fire predominated^9^ ^,^ ^25^ ^,^ ^27^ ^,^ ^29^ ^,^ ^32^, followed by manual pumping^23^ ^,^ ^34^ ^-^ ^36^ and automatic pumping^22^ ^,^ ^26^ ^,^ ^33^. Suction with fire is the traditional method used in China, however, there is a risk of burns^18^. Manual vacuum is created when using a suction pump. This method allows microcirculation to increase more effectively if compared to fire^18^. Finally, automatic pumping is created using an electric suction pump, which allows to adjust and measure the negative pressure inside the cup, being the most suitable method for scientific research^18^.

Only three studies^22^ ^,^ ^26^ ^,^ ^28^ reported the suction strength used, which should be standardized in the application protocols. The suction can be light (100 and 300 millibar/one or two manual pumpings), medium (300 and 500 milibar/three or four manual pumpings), strong (above 500 milibar/five or more manual pumpings) or pulsatile (pressure inside the cups is variable, between 100 and 200 milibar every 2 seconds)^47^ ^,^ ^49^. The medium suction is often indicated for painful conditions of the musculoskeletal system^18^.

There was also no standardization in relation to the application points of cupping therapy. Despite this, the application in specific acupoints in the cervical region, mainly on the bladder, gallbladder and small intestine meridians, prevailed^29^ ^,^ ^34^, and in the lumbar region on the bladder meridian^30^ ^-^ ^32^ ^,^ ^35^ ^-^ ^36^, followed by sensitive points^9^ ^,^ ^25^ ^-^ ^27^ ^,^ ^30^ named *Ashi* by TCM or trigger points by Western medicine.

Meridians are passages for the flow of “*qi*” (vital energy) and “*xue*” (blood), the two basic body fluids of TCM, which spread throughout the body surface, uniting the interior with the exterior of the body and connecting the internal organs, the joints and the extremities, transforming the whole body into a single organ^50^. Part of the meridians of the bladder, small intestine and gallbladder pass through the dorsal region. The acupuncture points are located in the meridians; besides local action, they also play a systemic action and reestablish the energy balance of the body by adjusting the function of the organs, maintaining homeostasis and treating the disease^51^, so the advantage in using them.

The trigger points or *Ashi* are specific points of high irritability; they are sensitive to digital pressure and can trigger local and referred pain^52^. They may be deriving from dynamic overload, such as trauma or overuse, or static overload, such as postural overloads occurring during daily activities and occupational activities^53^, besides emotional tension. Addressing these points can also be a way to relieve local pain^54^.

After the application of cupping therapy, both the acupoints of the meridians of the affected regions and the trigger points or *Ashi* may present bruising, erythema and/or ecchymoses. According to TCM, these signs represent stagnation of “*qi*” and/or “*xue*” and may help the therapist in identifying body disorders.

Finally, the meta-analysis revealed a significant reduction of the pain intensity score in adults with chronic back pain by using cupping therapy (p = 0.001). Compared with a control group (usual care/other intervention/waiting list), this modality has advantages in relieving pain, as can be seen in Figure 5.

Only two studies^24^ ^,^ ^30^ did not present a statistically significant difference between the groups on the benefit or harm of this intervention (Figure 5). In fact, the first study^24^ pointed out that cupping therapy has the same effect as other intervention (progressive muscle relaxation) in reducing chronic nonspecific neck pain; despite this, cupping therapy was better than relaxation in improving well-being and decreasing sensitivity to pressure pain. The authors^24^ justify this result, among other limitations, due to the fact that cupping therapy was performed by patients’ relatives or friends at home. The second study^30^, despite having found a positive result on the intensity of pain, did not obtain a result in the meta-analysis. It is believed that this may have been due to the fact that both groups received the intervention of soft cupping and both obtained positive results.

In the other studies^23^ ^,^ ^25^ ^-^ ^28^ ^,^ ^31^ ^-^ ^32^ ^,^ ^34^, the intervention reduced the probability of the outcome, being the study with the largest sample^31^ the one the most contributed (15.68% weight in the meta-analysis) for this (Figure 5). In fact, all these studies reported promising results of intervention on pain intensity.

However, the results of the effectiveness of cupping therapy still need to be confirmed by subgroup analyzes, based on different types of application techniques and control groups. In addition, it is important to perform meta-regression to find the source of heterogeneity of RCTs.

In a general way, the results showed a substantial variation in the application of cupping therapy, especially in relation to the type of technique, as well as differences in the control group, which made subgroup or meta-regression unfeasible, respectively, due to the small number of studies with each of these specifications.

## Conclusion

Cupping therapy is a promising method for the treatment and control of chronic back pain in adults, since it significantly decreases pain intensity scores when compared to control groups. However, the high heterogeneity and the median methodological quality of RCTs has limited the findings.

Despite this, a protocol can be established for this clinical condition: application of dry cupping technique in 5 sessions, with permanence of the disposable or plastic cups on the skin for about 8 minutes, preferably automatic or manual pumping, with medium suction strength, and three to seven days interval between applications. It is better to opt for acupoints of the dorsal region, especially those from the bladder meridian in the lumbar region, and for the meridians of the bladder, gallbladder and small intestine in the cervical and thoracic regions, as well as *Ashi* or trigger points. This protocol needs to be validated in future studies. And the main outcomes evaluated for this clinical condition were pain intensity, physical disability, quality of life and nociceptive threshold before the mechanical stimulus (pressure).
